# Does ventilatory assistance before umbilical cord clamping reduce the risk of early death or intraventricular hemorrhage in extremely preterm infants?

**DOI:** 10.1038/s41372-025-02258-5

**Published:** 2025-03-08

**Authors:** Kaquanta Barlow, Shoshana Newman-Lindsay, Evan Giusto, Anup Katheria, Satyan Lakshminrusimha

**Affiliations:** 1 Department of Pediatrics, UC Davis Children’s Hospital, Sacramento, CA USA; 2 D-5 Neonatal Units, UC Davis Children’s Hospital, Sacramento, CA USA; 3 Sharp Mary Birch Hospital for Women & Newborns, Sacramento, CA USA

**Keywords:** Developmental biology, Scientific community

## Manuscript citation

Fairchild, K. D., Petroni, G. R., Varhegyi, N. E., Strand, M. L., Josephsen, J. B., Niermeyer, S., Barry, J. S., Warren, J. B., Rincon, M., Fang, J. L., Thomas, S. P., Travers, C. P., Kane, A. F., Carlo, W. A., Byrne, B. J., Underwood, M. A., Poulain, F. R., Law, B. H., Gorman, T. E., Leone, T. A., … VentFirst Consortium (2024). Ventilatory Assistance Before Umbilical Cord Clamping in Extremely Preterm Infants: A Randomized Clinical Trial. JAMA network open, 7(5), e2411140. 10.1001/jamanetworkopen.2024.11140 [1].

## Type of investigation

Stratified randomized clinical trial.

## Research question

For extremely preterm infants, does providing ventilatory assistance during delayed umbilical cord clamping (DCC) reduce intraventricular hemorrhage (IVH) or early death? This study was based on improved hemodynamic stability observed in preterm lambs that were ventilated during DCC compared to lambs with cord clamping followed by ventilation [2].

## Methods

### Design

Multicenter, international, prospective, parallel-stratified, randomized clinical trial conducted from September 2016 through February 2023.

### Blinding

Clinicians at delivery were not blinded to the study arm. Radiologists who interpreted the head ultrasonography were blinded to the assigned study arm.

### Setting

The study was conducted at 12 perinatal centers across the United States and Canada.

## Participants

### Inclusion criteria

Women expected to deliver infants between 23 0/7 to 28 6/7 weeks gestation by best obstetric estimate.

### Exclusion criteria

Exclusion criteria included monochorionic twin and higher-order multiple pregnancies, medical emergencies requiring immediate delivery, known major fetal anomalies, severe fetal anemia, hydrops fetalis, decision not to pursue intensive care for the infant, or decision by the neonatologist or obstetrician to exclude participation due to maternal or fetal concerns.

### Randomization

**Fig. 1:**
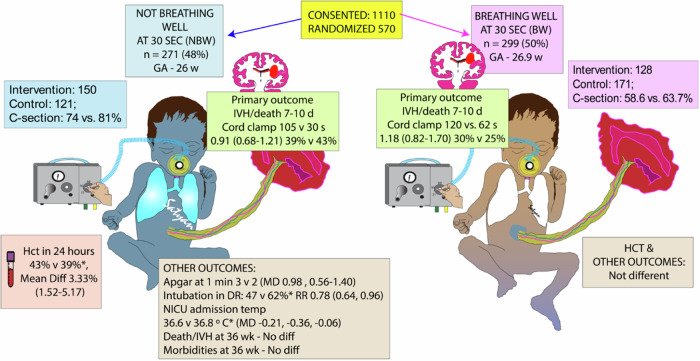
Graphic abstract of the Fairchild et al. “VentFirst” trial. GA gestational age at birth, NBW not breathing well, BW breathing well, IVH intraventricular hemorrhage, DR delivery room, RR relative risk, MD mean difference, Hct hematocrit, PMA postmenstrual age, Copyright Satyan Lakshminrusimha.

Mothers were randomized when delivery was thought to be imminent. Stratified block randomization by study site and GA cohort (23 0/7 to 25 6/7 weeks or 26 0/7 to 28 6/7 weeks, Fig. 1).

### Intervention

Infants received tactile stimulation and airway suctioning as needed during the first 30 s of DCC. Respiratory support was initiated 30 s after birth for infants in the intervention group; continuous positive airway pressure (CPAP) if breathing well or positive pressure ventilation (PPV) if not breathing well. Goal umbilical cord clamp time was 120 s.

### Control

In the control group, the umbilical cord was clamped at 30 s if the infant was not breathing well or 60 s if the infant was breathing well.

### Consent

All participating centers’ institutional review boards approved the protocol. Written informed consent was obtained from participating mothers.

## Outcomes

### Primary outcome

The primary outcome was death before day 7 or IVH of any grade on ultrasound obtained 7-to-10 days after birth.

### SecondaRY OUTComes

Secondary outcomes included 5-min Apgar score less than 5, lowest hematocrit in the first 23 h, medication for hypotension in the first 24 h, number of red blood cell transfusions in the first 10 postnatal days, death before 36 weeks corrected postmenstrual age, and severe brain injury (defined as grade 3 or 4 IVH, cerebellar hemorrhage, or cystic periventricular leukomalacia) [1].

### Analysis and sample size

Sample size calculation was based on an estimate that 37% of the cohort would be in the not-breathing-well group at 30 s of life, with stratification by GA. A sample of 940 infants would result in 80% power to detect an odds ratio of 0.5 for intervention compared to control in the not-breathing-well cohort.

The primary analysis plan was intention to treat, with Cochran-Mantel-Haenszel tests to compare binary predictor and outcome variables with stratification. The analyses were stratified by GA category and site. Sites with fewer than 25 participants were grouped together. The study excluded infants for whom the duration of DCC diverged from the prescribed time by more than 15 s.

## Main results

570 infants were included in the analysis, and the protocol was initiated in 475 infants (83.3%). Median cord clamping time was 120 s (IQR, 64–124) in the intervention group and 60 s (IQR, 30–63) in the control group.

## Primary outcome

The incidence of IVH on 7-to-10-day head ultrasonography or death prior to day 7 was not significantly different between the intervention group and control group (34.9% vs. 32.5%; adjusted relative risk-RR 1.02, 95 percent confidence interval [CI 0.81–1.27]). When the infants were split into not-breathing-well and breathing-well cohorts, there was no significant difference in the incidence of IVH or death (Fig. 1).

## Secondary outcomes

Infants in the intervention group of the not-breathing-well cohort had a higher lowest median hematocrit in the first 24 h after birth (median 43% IQR 37–47%) compared to the control group (median 39% IQR 34–43%).

Infants in the intervention group of the not-breathing-well cohort had higher 1-min Apgar scores, were less likely to require endotracheal intubation, but lower NICU admission temperatures (36.6 °C vs. 36.8 °C, Fig. 1). There were no significant differences in secondary outcomes in the “breathing-well” cohort.

## Study conclusions

In extremely preterm infants, ventilatory assistance starting at 30 s with cord clamping at 120 s, compared to cord clamping at 30 to 60 s after birth followed by ventilatory assistance, did not impact the occurrence of IVH at age 7-to-10 days or death prior to day 7.

## Commentary

In extremely preterm infants with poor respiratory effort, there may be a delay in postnatal increase in pulmonary venous return. Umbilical venous return during DCC remains an important source of left ventricular preload as the preterm infant is establishing respirations. Optimal lung inflation with CPAP or PPV, when the preterm infant is still attached to the placental circulation, should enable a more stable transition from the fetal to neonatal period and potentially reduce the risk of IVH and early death.

This premise is based on a lamb study demonstrating that clamping the umbilical cord once the lung is aerated avoids decreases in heart rate, ventricular output, and carotid flow [2, 3]. Lambs were delivered by cesarean section under general anesthesia and typically lacked spontaneous respirations. The Clamp-First group of lambs were deprived of any PPV for 2.1 ± 0.1 min after cord clamping. In contrast, the Vent-First lambs had the advantage of DCC and PPV for 3.7 ± 0.3 min before clamping the cord [4]. A different lamb study with a shorter gap between immediate cord clamping and PPV initiation did not demonstrate any hemodynamic benefit [5, 6]. These methodological and species differences can partly explain the lack of benefit demonstrated in the clinical VentFirst trial [7]. The authors speculate that the lack of difference in the primary outcome could be due to a lack of assessment of lung aeration in the study design and other “physiologic perturbations”. While spontaneous breathing enhances umbilical venous return during DCC [8], it is unclear whether PPV has a similar effect.

Because pulmonary venous return may be already established at 60 s of DCC, it is unlikely that respiratory support during 120 s of DCC would provide added benefit [9, 10]. The VentFirst study did not show any benefit in IVH/death or hematocrit at 24 h with respiratory support during DCC in the breathing well group [1]. These data suggest that in spontaneously breathing preterm infants, additional respiratory support does not reduce the risk of IVH or increase hematocrit. Preterm infants who are breathing well by 30 s during DCC have a higher survival without IVH compared to infants not breathing well during DCC [1].

Among preterm infants not breathing well, the intervention arm provided CPAP/PPV during a more prolonged period of 120 s of DCC. The higher Apgar score at 1 min, and higher hematocrit at 24 h are likely the result of DCC during respiratory support.

The lower need for intubation in the intervention arm may be due to a higher denominator (number of infants deemed not breathing well in the intervention arm) rather than the intervention itself. The percentage of neonates not breathing well at 30 s was higher (47.5%) than expected (37%). Additionally, more infants were assessed to be “not breathing well” in the intervention arm compared to the control arm (150/278 – 54% vs. 128/292 – 44%). This difference was marked in the lower gestational age stratum of 23–25 weeks compared to 26 to 28 weeks (71/116 – 61% vs. 45/115 – 39%).

## EBM lesson: goals of randomization

Randomization is used to assign participants into treatment and control groups. The goal of randomization is to ensure that both measured and unmeasured baseline characteristics that could affect an observed association will be distributed similarly among the groups [11]. This study used stratified block randomization [12]. The VentFirst investigators used block sizes varying from two to six. Randomization was stratified by site and gestational age.

Block randomization ensures a relatively equal number of participants in each group throughout the trial and helps to remove temporal biases. Blocks of a pre-specified size have equal numbers in intervention and control groups. To reduce bias in both blinded and unblinded trials, the size of the blocks is varied randomly. A limitation to block randomization is that participants with secondary diseases could be unbalanced between blocks.

Stratified randomization balances the distribution of variables that predict outcome to ensure they are approximately equal in the intervention and control groups. The specific variables must be decided by the researcher based on potential effect of each covariate. Additionally, stratification is important in a multisite trial because it helps to balance patient characteristics and should decrease bias due to differences between populations at different sites. Furthermore, stratified analysis can be used to assess for effect modification and whether a specified outcome differs across subgroups. A limitation of stratification is that only a few baseline variables can be accounted for in this way due to the increasing complexity of randomization schemes that account for all stratified variables. The decision of whether to stratify based on important baseline characteristics has been debated in the literature and remains unresolved [13].

## Summary

This trial established the safety and feasibility of intact cord respiratory support. Using stratified block randomization, infants at 23–25- and 26–28-weeks’ gestation were enrolled. Different time-based clamp times were achieved in both groups. There was no difference in the primary outcome. Results from several ongoing studies evaluating intact cord ventilation with prolonged clamp tines are needed before broad adoption of CPAP or PPV during DCC for preterm infants. In the meantime, stimulation of preterm infants to support spontaneous breathing during DCC may be beneficial.
